# Acute Liver Failure in a Healthy Young Female With COVID-19

**DOI:** 10.1097/PG9.0000000000000108

**Published:** 2021-07-22

**Authors:** Babak John Orandi, Geling Li, Deepti Dhall, Prachi Bajpai, Upender Manne, Nitin Arora, Ailing Lu, Ana Carolina Coronado, Rachel Kassel, Swetha Pinninti, Cora Elizabeth Lewis, Christopher Chapleau, Jayme Elizabeth Locke, Luz Helena Gutierrez Sanchez

**Affiliations:** From the * Department of Surgery, University of Alabama at Birmingham, Birmingham, AL; † Department of Pathology, University of Alabama at Birmingham, Birmingham, AL; ‡ Division of Neonatology, Department of Pediatrics, University of Alabama at Birmingham, Birmingham, AL; § Division of Pediatric Gastroenterology, Hepatology and Nutrition, Department of Pediatrics, University of Alabama at Birmingham, Birmingham, AL; ‖ Division of Pediatric Infectious Diseases, Department of Pediatrics, University of Alabama at Birmingham, Birmingham, AL; ¶ Department of Epidemiology, University of Alabama at Birmingham, Birmingham, AL; # Department of Pharmacy, University of Alabama, at Birmingham, Birmingham, AL.

**Keywords:** COVID-19, acute liver failure, liver transplant

## Abstract

Several well-described manifestations of infection with severe acute respiratory syndrome coronavirus 2 (SARS-CoV-2) have been reported. Among them, a transient elevation of liver enzymes is the typical presentation of coronavirus disease 2019 (COVID-19) liver-related injury. The mechanism of liver involvement is likely a combination of viral injury and immune-mediated inflammation. In contrast, acute liver failure in the setting of COVID-19 has rarely been reported. Herein, we report a case of pediatric acute liver failure in a previously healthy female adolescent infected with SARS-CoV-2 with biopsy evidence of replicating virus in hepatocytes, which has not been previously reported.

## INTRODUCTION

Most symptomatic cases of severe acute respiratory syndrome coronavirus 2 (SARS-CoV-2), the causative agent of the coronavirus disease 2019 (COVID-19) pandemic, typically present with fever and respiratory symptoms. Elevated liver enzymes are seen in 14-78% of cases (1). Acute liver failure (ALF) with concurrent COVID-19 has been reported in a few cases. Herein, we report a case of pediatric ALF ostensibly precipitated by COVID-19 in an otherwise asymptomatic patient with biopsy findings demonstrating replicating SARS-CoV-2 RNA in hepatocytes.

## CASE REPORT

A nonobese 15-year-old girl with history of menorrhagia, presented to an outside hospital with 3 days of anosmia, ageusia, 2 days of nausea, vomiting, and the absence of pulmonary symptoms. She had a confirmed recent SARS-CoV-2 exposure at school. She denied any medications, drugs, alcohol, or tobacco, except initiation of norethindrone acetate 1.5 mg and ethinyl estradiol 30 µg for menorrhagia 7 weeks before presentation.

**FIGURE 1.**
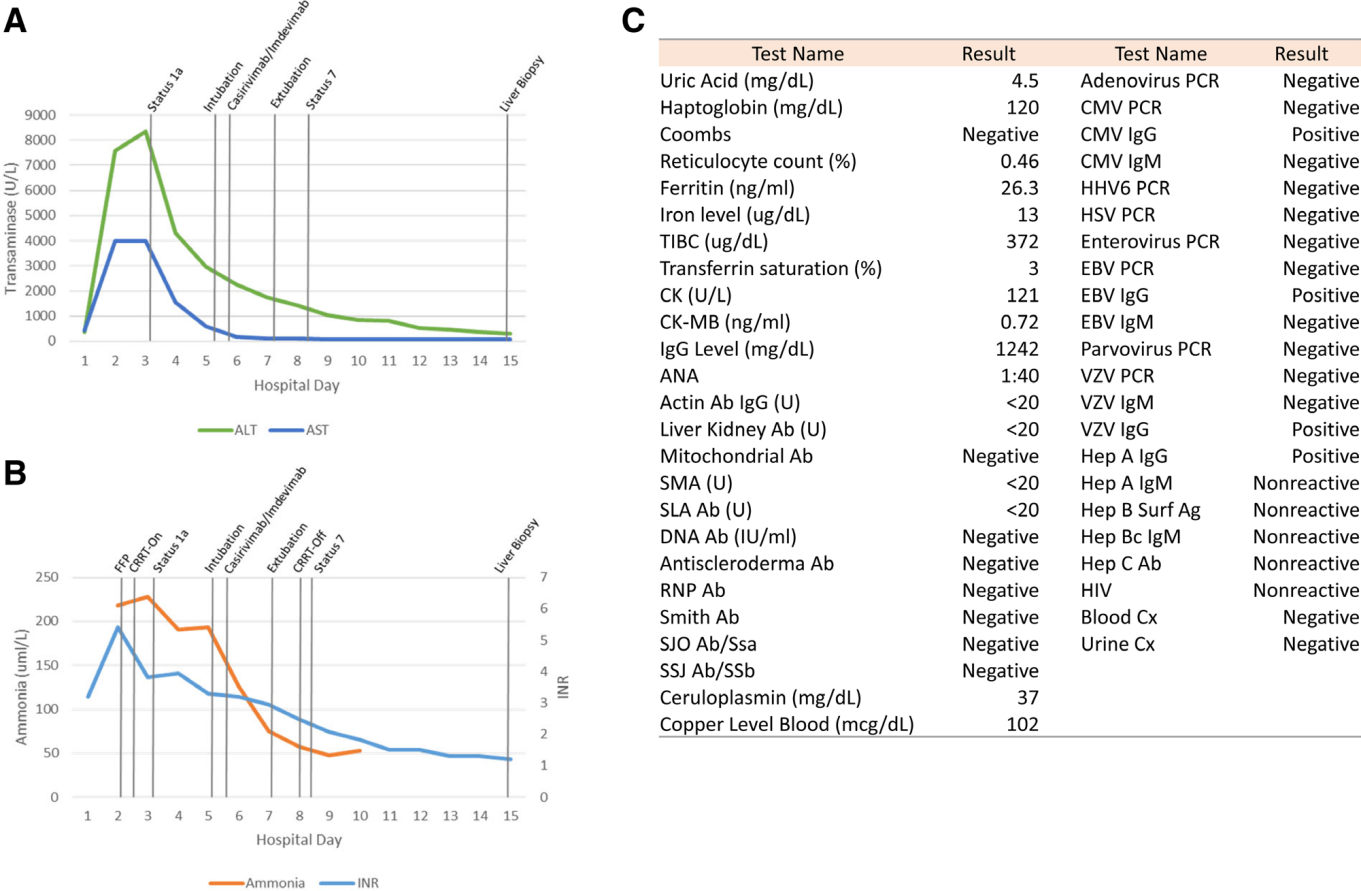
A) ALT and AST trend during the patient’s hospitalization, mean laboratory value was plotted for hospital days with multiple results. B) International normalized ratio (INR) and ammonia levels during the patient’s hospitalization, mean laboratory value was plotted for hospital days with multiple results. C) Laboratory work-up obtained to evaluate the patient’s acute liver failure etiology. ALT = alanine aminotransferase; AST = aspartate aminotransferase; CRRT = continuous renal replacement therapy; FFP = fresh–frozen plasma.

The patient transferred to our facility for dehydration, anemia, and transaminitis (Fig. 1A). On arrival, she was hemodynamically stable and neurologically intact. Initial testing demonstrated worsening transaminitis (Fig. 1A) and SARS-CoV-2 positivity by reverse transcription-polymerase chain reaction test (RT-PCR) from the respiratory tract. She developed ALF on hospital day (HD) 2 (Fig. 1A, B). Extensive testing to determine ALF etiology was unrevealing (Fig. 1C).

We initiated treatment with vitamin K, broad-spectrum antibiotics, lactulose, rifaximin, and dexamethasone. Continuous renal replacement therapy (CRRT) was initiated the evening of HD2 to manage hyperammonemia.

On HD3, she was listed with status 1A priority for liver transplant. N-acetyl cysteine was initiated. On HD5, she was intubated for worsening mental status. Head CT was normal. Because of concerns regarding how COVID-19 might affect her if she required liver transplant and immunosuppression, we applied for and received an emergency compassionate use investigational new drug request for casirivimab/imdevimab (REGN10933/REGN10987; Regeneron, Tarrytown, NY), an admixture of recombinant monoclonal antibodies to the receptor-binding domain of the COVID-19 virus’s spike protein. On HD5, she received 2.4 g without adverse effects. ALF precluded safe use of remdesivir.

On HD6 the patient demonstrated improvement in coagulopathy, hyperammonemia and mental status. She was extubated on HD7, and CRRT was discontinued on HD8.

**FIGURE 2.**
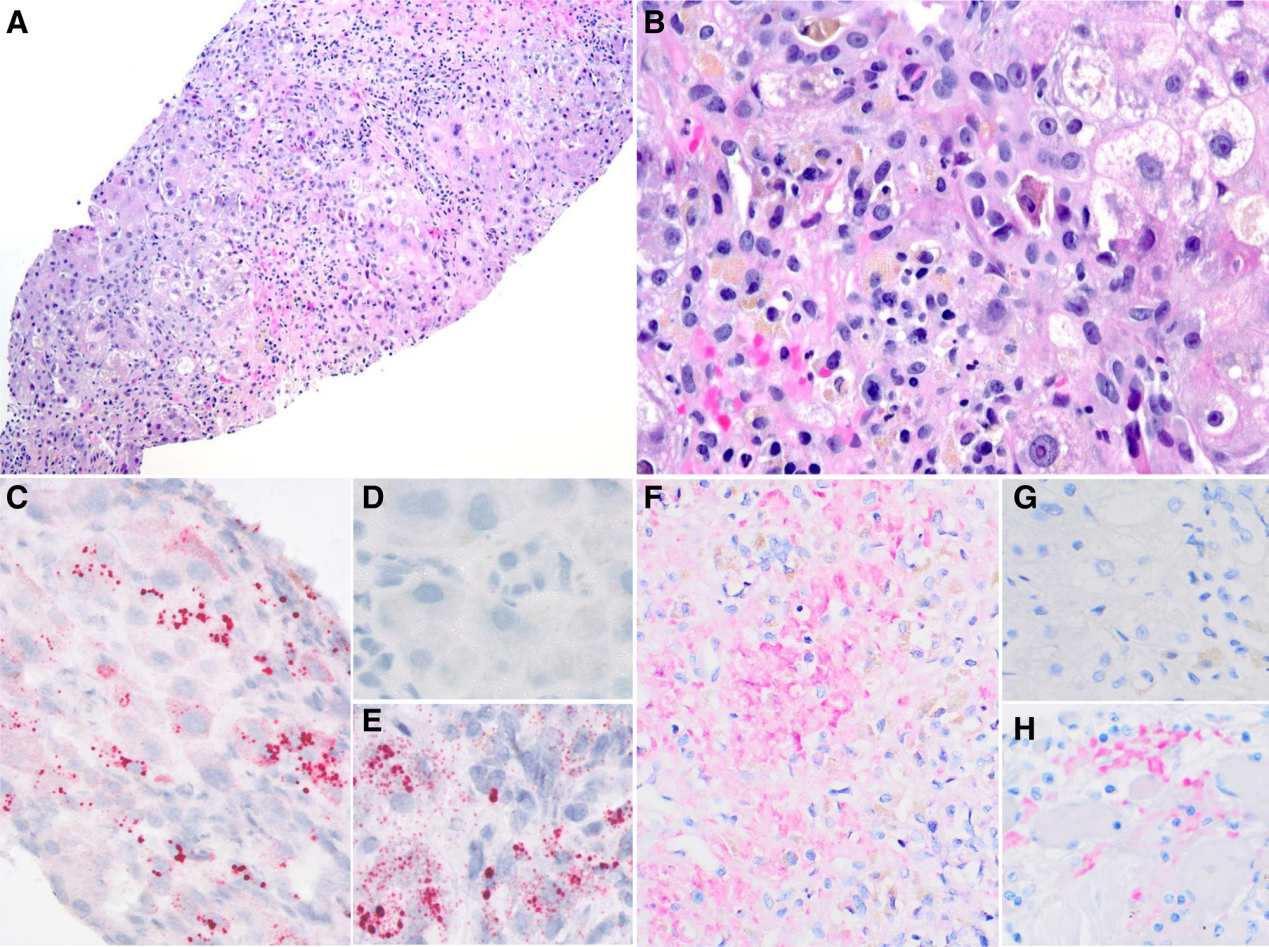
Liver biopsy showing acute hepatitis pattern of injury with submassive necrosis and canalicular cholestasis associated with SARS-COV-2 replicating virus. A) and B) Liver showing prominent necrosis involving centrilobular zones with prominent mixed inflammatory cell infiltrate including lymphocytes, plasma cells and neutrophils, and residual foci of hepatocytes. The residual hepatocytes show prominent nucleoli, ballooning degeneration, scattered apoptosis, pseudoacini formation, and mild steatosis. Hematoxylin-eosin (H&E) stain; Original magnification ×100 (A) and ×400 (B). C) In situ-hybridization with a V-nCOV2019—orf1ab—sense probe detecting replicating SARS-COV-2 RNA (red chromogen) in hepatocytes. A negative control using a probe targeting DapB (Bacillus subtilis strain) (D) and a positive control using a probe targeting the house-keeping gene POLR2A (red chromogen) (E) are shown. F) Immunohistochemistry of liver tissue using a rabbit anti-SARS-COV-2 nucleocapsid protein antibody (red/pink chromogen; AB273167) confirming SARS-COV-2 nucleocapsid protein expression. A negative isotype control (G) and a positive control using an autopsy lung tissue infected with SARS-COV-2 (H) are shown.

Liver biopsy on HD15 showed an acute hepatitis pattern of injury with predominantly centrizonal areas of confluent necrosis and lobular cholestasis. Necrotic areas were focally hemorrhagic and showed mild lymphoplasmacytic infiltrate with admixed histiocytes, few eosinophils, and neutrophils. Portal inflammation was minimal and interlobular bile ducts were largely intact. No fibrin thrombi were identified. Residual hepatocytes showed ballooning degeneration, some with prominent nucleoli, apoptosis, pseudoacini formation, and mild steatosis (<10%) (Fig. 2A, B). In-situ hybridization assay was performed using RNAscope Probe-nCoV2019-orf1ab-sense-C2 (cat.no. 859151) on 5-μm paraffin-embedded sections of liver tissue according to manufacturer instructions (Advanced Cell Diagnostics, Inc Newark, CA). In situ-hybridization detected replicating SARS-COV-2 RNA in hepatocytes (Fig. 2C). Immunostaining confirmed expression of SARS-COV-2 nucleocapsid protein (Fig. 2F).

The patient discharged on HD16. She is doing well 5 months postdischarge with normal liver test.

## DISCUSSION

Pediatric ALF is a life-threatening condition with a broad differential diagnosis. Our patient’s extensive evaluation was negative except for SARS-CoV-2. ALF in the setting of COVID-19 has only been described in a handful of cases, and only one other case had a liver biopsy. In that case, a 35-year-old woman with COVID-19 infection and systemic lupus erythematosus underwent biopsy, which showed findings consistent with acute hepatitis, and no viral inclusions (neither SARS-CoV-2 tissue PCR nor immunohistochemistry were available) (2).

Although SARS-Cov-2 infection contributes to acute liver injury, it is often mild. Our liver pathology knowledge mostly originates from examining post-mortem liver biopsies from patients with severe pulmonary COVID-19 disease. In those patients, the liver often showed mild acute hepatitis, hepatic apoptosis, mild portal inflammation, and macrovesicular steatosis (3–6). SARS-CoV-2 PCR of liver tissue was positive in approximately half of patients, and its positivity was not significantly associated with aspartate aminotransferase/alanine aminotransferase elevation (5). It was unclear if SARS-CoV-2 damages the liver through direct viral-mediated injury, an immune-mediated inflammatory response, or a combination. Direct evidence of hepatocellular infection by SARS-Cov-2 is lacking. In 2 adult COVID-19 patients with massive hepatic apoptosis and pulmonary diffuse alveolar damage, typical coronavirus particles, characterized by spike structures, were identified in the cytoplasm of hepatocytes by transmission electron microscopy (3). Our patient’s biopsy showed submassive hepatocellular necrosis involving the centrilobular zone and minimal portal inflammation. In situ-hybridization with a probe specific for the orf1ab gene of the SARS-CoV-2 negative-sense RNA (replicative intermediate) demonstrated replicating SARS-COV-2 RNA in hepatocytes. Immunostaining confirmed the expression of SARS-COV-2 nucleocapsid protein. Acute hepatitis caused by asymptomatic SARS-CoV-2 infection is rarely reported; infection of liver tissue by SARS-CoV-2 has not previously been confirmed (7). To our knowledge, this is the first case describing SARS-CoV-2 viral-mediated submassive liver necrosis, in a patient with minimal extrahepatic symptoms of COVID-19 infection, determined by replicating RNA and expression of the nucleocapsid protein of SARS-CoV-2.

Another unique aspect of this case is use of monoclonal antibodies to the receptor-binding domain of the SARS-CoV-2 virus spike protein. We noted a temporal association with administration of these monoclonal antibodies and significant improvement in the patient’s clinical course, as evidenced by improvements in aspartate aminotransferase, alanine aminotransferase, and international normalized ratio, though these laboratory values showed some improvement for the first time 12–18 hours before medication administration. There is a striking reduction in SARS-CoV-2 viral load within 48 hours of administration of these monoclonal antibodies (8); however, we cannot definitively determine a causal relationship between the use of these antibodies and clinical improvement.

We present a pediatric ALF case with a negative etiologic evaluation except for a positive RT-PCR test for SARS-CoV-2, and absence of pulmonary symptoms. In-situ hybridization and immunohistochemistry findings on liver biopsy showed SARS-CoV-2 replication. Although these findings do not prove causality, and no studies have been performed to determine the presence of SARS-CoV-2 in liver tissue of infected patients who are otherwise asymptomatic, the presence of viral particles in the liver tissue of this case raises the possibility of COVID-19-induced ALF. Thus, vigilance is warranted for SARS-CoV-2-related liver injury that progresses to ALF.

## ACKNOWLEDGMENT

We would like to thank the patient and the patient’s mother for agreeing and granting us informed consent for publication of the case details. Informed consent was documented within the patient’s medical record.
